# Risk Assessment of Heavy Metal in Farmlands and Crops Near Pb–Zn Mine Tailing Ponds in Niujiaotang, China

**DOI:** 10.3390/toxics11020106

**Published:** 2023-01-22

**Authors:** Qinyuan Li, Zhiwei Han, Yutong Tian, Han Xiao, Miao Yang

**Affiliations:** 1 College of Resources and Environmental Engineering, Guizhou University, Guiyang 550025, China; 2 Key Laboratory of Karst Georesources and Environment, Ministry of Education, Guizhou University, Guiyang 550025, China

**Keywords:** lead-zinc tailings, heavy metal, farmland, crops, soils, risk assessment

## Abstract

To accurately evaluate the pollution and risk of heavy metals in crops and farmlands near mines, we determined the contents of Cr, Ni, Cu, As, Cd, Pb, and Zn in 10 farmland soil sampling sites and six crops (pak choi, rice, spring onion, radish, Chinese cabbage, *Chrysanthemum coronarium*) in an area near the Niujiaotang Pb–Zn mine in Duyun City, China. Four evaluation methods were compared, including the potential ecological hazard index, Nemeiro comprehensive pollution assessment, risk assessment code, and the ratio of secondary phase to primary phase methods. The average concentration of As, Cd, Pb, and Zn exceeded the soil environmental background levels in Niujiaotang and Guizhou Province. Cd exceeded the standard substantially, and Zn pollution accumulation was the most evident. Heavy metal contamination of crops was in the order pak choi > Chinese cabbage > spring onion > paddy > radish > *Chrysanthemum coronarium*, whereas heavy metal concentration in crops were in the order Zn > As > Cr > Cd > Ni > Pb. The levels of all heavy metals except Cu exceeded Chinese food hygiene standards. Carcinogenic and non-carcinogenic chemicals in crops present significant risks to adults and children. Risk evaluation considering the morphological contents of heavy metals rather than their total concentration was more accurate for environmental quality assessment of agricultural soils. Samples should be collected at different times to study the spatial and temporal distribution, and further studies on the migration transformation of heavy metals between the tailings pond-soil-crop should be conducted.

## 1. Introduction

China is one of the world’s largest producers and consumers of Pb and Zn. The cumulative production of Pb and Zn in China until 2007 was approximately 6.69 and 12.59 Mt, respectively, and approximately 1.62 Mt of Pb and 3.32 Mt of Zn were released into the environment due to mining, processing, and smelting activities. During mining and smelting, heavy metals can be transported to the surrounding environment through atmospheric deposition, surface water contamination (including acid mine drainage), and groundwater seepage, thereby posing serious ecological risks [1,2]. Guizhou Province is famous for its rich mineral resources and long history of nonferrous metal mining and smelting [3]. Despite the economic benefits of mining, this activity causes heavy metal pollution of nearby soils and crops. Heavy metal pollution is insidious, long-term, and irreversible, and heavy metals that enter the soil cannot be degraded and thus, they persist in the soil for a long time and accumulate, eventually causing harm to the ecological environment and human health via food chain contamination [4,5].

The Niujiaotang deposit is located in the north-south tectonic deformation zone of Guiding at the southwest edge of the Yangzi Plate. It is a Cd-rich Zn deposit and has the highest Cd content in Guizhou Province [6]. The Pb–Zn mine tailings in Niujiaotang are large in volume and mostly stored in constructed tailing ponds. They are mainly composed of gray, off-white, and gray-brown Pb–Zn fine-grained tailings [7]. During the natural accumulation of tailings, influenced by atmospheric rainfall, the tailings sand will undergo epigenetic geochemical reactions with carbon dioxide and oxygen in the air in the water–rock system, which activates the heavy metals in the tailings sand [8,9]. The Cd content in the Niujiaotang Pb–Zn ore in Duyun, Guizhou is unusually high, generally ranging from 2284 to 9850 μg/g, with a maximum of 13,400 μg/g, and its reserves have reached the scale of large deposits (medium-large Zn and Cd reserves greater than 5000 t [10]. Sun et al. analyzed the contamination characteristics of different heavy metals (Pb, Zn, Cr, Cu, Cd, and Hg) and the Pb isotopic composition in natural soil (layers A and C) in the Shuikou Mountain Pb–Zn mining area (and nearby areas) of Hunan, a typical Pb–Zn mining area in China. Their results showed that the soil in layer A was clearly contaminated with heavy metals owing to the influence of Pb–Zn beneficiation and smelting activities. Pan et al. reported that the Niujiaotang Cd-rich Zn deposit is a polymetallic sulfide deposit with high Zn and Cd contents in its ore.

In recent years, heavy metal contamination of agricultural soils and crops near mining areas and the associated risks have attracted widespread attention from various sectors and researchers worldwide. It is important to analyze and understand the morphological characteristics of heavy metals in the soil to identify their sources and determine their biological effectiveness [11]. The migration capacity of heavy metals is closely related to their mobile forms, which affect their cycling and ecotoxicity [12]. For instance, heavy metals in the exchangeable state have high mobility and are easily absorbed by organisms, and this is the most unstable and toxic form, which causes direct toxicity. The reducible and oxidizable states can be transformed into biologically effective forms in reducing and oxidizing environments, respectively, and their potential harm to the environment is known as potential toxicity. The residual state of heavy metals is usually inert and does not participate in chemical reactions, does not change with modifications in the external environment, and has low bioavailability [13]. The results of this study indicated that the total content of heavy metals in the soil does not completely determine their environmental behaviors and biomorphic effects, and plants do not fully absorb and utilize all heavy metals from the soil, only forms in the biologically effective state (mainly the exchangeable and carbonate-bound states) are absorbed. In contaminated areas, the harm to humans upon consumption of heavy metal-contaminated rice is even higher than that caused by ingestion of heavy metal-contaminated water, and long-term consumption of the former can easily lead to chronic poisoning [14].

In this study, we aimed to elucidate the distribution of heavy metals in farmlands and crops near the Duyun Niujiaotang Pb–Zn mine through sampling and experimental analysis. The pollution level, pollution risk, and crop health risk were evaluated based on the total concentration and distribution of heavy metals. We expect our results to contribute to risk assessments of soils and crops near mines and the development of prevention and mitigation measures to ensure food security, human health, and the sustainability of mining areas.

## 2. Materials and Methods

### 2.1. Study Area

The Niujiaotang Cd-rich Pb–Zn deposit is located in Duyun City, Guizhou Province (latitude: 26.226643; longitude: 107.666317), which has a subtropical monsoon humid climate, moderate cold in winter and a coldest average daily temperature of 5.6 °C in January. There is no severe summer heat, and the average daily temperature in the hottest month (July) is 24.8 °C. Rainfall is heavy, with an annual average of 1431.1 mm. Rain and heat occur in the same season. The annual average temperature is 16.1 °C and the frost-free period is approximately 300 days. The soil in the study area is generally severely polluted with heavy metals such as Zn and Pb and the heavy metals in the main agricultural products substantially exceed the standards, posing a serious health threat to nearby residents.

According to a preliminary investigation, Bagu town has rich mineral resources, and the main known deposits are of Pb–Zn, limonite, and barite. The Fanjia River Pb–Zn ore in the Mapo Pb–Zn section with shallow metal reserves of 500,000 t belongs to the national medium-sized deposits, with grades of 0.84–11.3%, whereas the geological reserves of the Pb–Zn ore in the Qingshui pond section and Duniu Pb–Zn section amount to approximately 200,000 t, with grades of 0.57–10.8%. There are nine processing plants and 34 mining holes in the town, with a daily output of 20,000 t of ore, producing 0.08 million t of concentrated powder. The currently known reserves of this deposit are more than 350,000 t of Zn (Zn grade of 5.85–24.48%) and 5299.14 t of Cd (large scale or above) [6].

### 2.2. Sample Collection and Preprocessing

**Figure 1:**
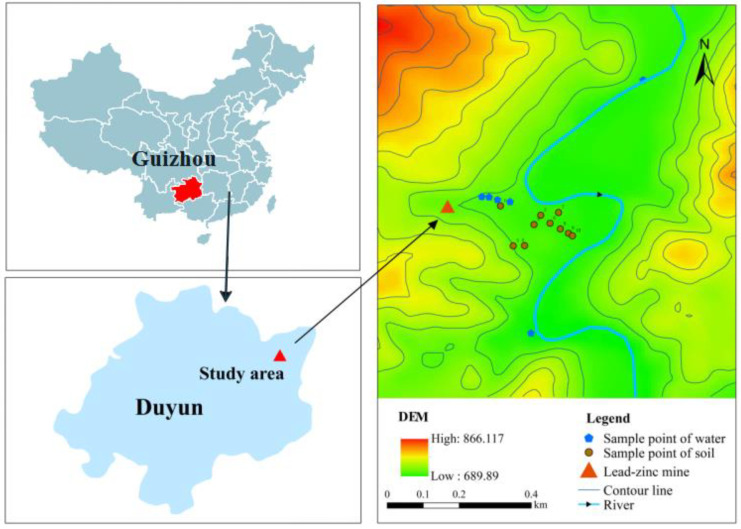
Map of the study area and sampling locations.

The sampling points were located along the Caiyuan River in farmland areas near the Pb–Zn mine, and the samples were collected during an abundant water period (August 2021) and a flat-water period (November 2021). In total, seven surface water samples and 10 crop samples (including pak choi, rice, spring onion, radish, Chinese cabbage, and Chrysanthemum coronarium) were collected, along with 10 inter-root soil samples. When collecting soil samples, a 3 × 3 m² area was selected and five soil samples were taken by the plum method and mixed into one point sample. Ultrapure water was used in the experiments, and all reagents were of analytical grade. The crop and soil samples were placed into self-sealing bags and water samples were collected into polyethylene bottles, which were all transported to the laboratory. To prepare the samples, the crop samples were washed with tap water after manual shaking to remove large dirt particles on the surface. The crop samples were divided into four parts: leaves, stems, roots, and fruits. All samples were rinsed with deionized water and placed into an oven at 105 °C for 30 min, dried at 75 °C to constant weight, crushed, ground through a 100-mesh sieve, and then set aside. Subsequently, we removed stones, biological debris, and plant fragments from the soil samples, air-dried them in a cool and dry environment, ground them, and passed them through 60-mesh and 200-mesh sieves. Finally, the samples were then bagged and sealed. The surface water samples were filtered through a 0.45-μm membrane, acidified with ultrapure nitric acid to pH < 2 and stored in sealed polyethylene plastic bottles at 4 °C until analysis. The pH, Eh, and EC were measured by Multi3630, WTW, Germany. Reagent blanks, three parallel soil samples and the national standard soil sample GSS-37 were used for quality control during the analysis. The correlation coefficients of the calibration curves of the heavy metal standard solutions were all greater than 0.999, and the experiments were conducted in three replicates. The model of the oven was DHG-9140A produced by Shanghai Huitai Instrument Manufacturing Co. The limits of detection (LODs) were 0.01 mg L^−1^ for Cd, 0.001 mg L^−1^ for As, 0.005 mg L^−1^ for Pb, 0.005 mg L^−1^ for Ni, 0.02 mg L^−1^ for Zn, 0.01 mg L^−1^ for Cu, and 0.005 mg L^−1^ for Cr. The sampling locations are shown in Figure 1.

### 2.3. Sample Analysis

Nitric acid-hydrofluoric acid was added to the soil samples, ratio 3:1 (*v*/*v*) for high-temperature digestion. The concentrations and chemical forms of heavy metals (Cr, Ni, Cu, As, Cd, Pb, and Zn) in soil and crop solutions were determined by inductively coupled plasma-mass spectrometry (ICP-MS). A procedural blank, parallel experiment, and national standard soil sample (GSS-37) were used for quality control during the analytical process. Ultrapure water was used in the experiments, and all reagents were of analytical grade. The vessels were soaked in 20% HNO3 solution for more than 24 h, washed with ultrapure water, and then dried.

Determination of the chemical forms of heavy metal elements is an important basis for the study of cyclic patterns such as elemental migration and transformation. The extraction steps were conducted according to the sequential extraction scheme of the European Community Standards Agency (BCR sequential extraction procedure), as follows. The samples were sequentially leached under acetic acid solution (CH_3_COOH) to isolate the weak acid-extracted state (F1), hydroxylamine hydrochloride solution (NH_2_OH·HCl) to isolate the reducible state (F2), and ammonium acetate (NH_4_Ac) to isolate the oxidizable state (F3). The total concentration of heavy metals and the contents of the first three forms were determined by ICP-MS (Thermo Fisher). The sum of the first three forms was subtracted from the total concentration to obtain the content of the residual state, which was denoted as F4.

### 2.4. Pollution and Ecological Risk Assessment

There are numerous methods to evaluate soil heavy metal risk, the most robust and widely used being the index evaluation method [15]. This method is based on the total concentrations of heavy metals, and it can visually reflect the correlation between measured and background concentrations of heavy metals to evaluate their risk in the soil. According to their evaluation criteria, index evaluation methods can be divided into single pollution [16], Nemeiro [17], geological accumulation [18], and potential ecological risk index methods [19]. Ecological risk evaluation, which is based on the total amount of soil heavy metals, often does not effectively represent the chemical activity and bioavailability of heavy metals. In contrast, evaluation based on the morphology of heavy metals can more realistically predict the ecological risk of soil heavy metals and provide a more scientific basis for pollution prevention and control [20]. Therefore, morphology-based evaluation methods, including the commonly used risk assessment code (RAC) method [21] and ratio of secondary phase to primary phase (RSP) method [22], have become more popular. To select the method that can most objectively and comprehensively reflect the pollution level and ecological risk of soil heavy metals, different evaluation methods should be compared, and the environmental effects and behavioral characteristics of heavy metals should be comprehensively evaluated.

#### 2.4.1. Potential Ecological Risk Index (RI) Method

The potential ecological RI method is an internationally recognized method widely used for the evaluation of soil heavy metal pollution [23]. It combines environmental chemistry, biotoxicology, and ecology to quantitatively classify the degree of potential heavy metal hazard [24]. The *RI* can be calculated as follows:

(1) $$\begin{array}{c} \;C_{f}^{i}=\frac{C_{s}^{i}}{C_{n}^{i}\;}\; \end{array}$$

(2) $$\begin{array}{c} E_{r}^{i}=T_{r}^{\;i}\times C_{f}^{i} \end{array}$$

(3) $$\begin{array}{c} RI=\overset{n}{\underset{i=1}{\sum }}E_{r}^{i}=\overset{n}{\underset{i=1}{\sum }}T_{r}^{\;i}\times C_{f}^{i}=\overset{n}{\underset{i=1}{\sum }}T_{r}^{\;i}\times \frac{C_{s}^{i}}{C_{n}^{i}\;}, \end{array}$$

where *C^i^_f_* is the contamination coefficient of heavy metal *i* relative to the reference value; *C^i^_s_* is the measured concentration of heavy metal *i*; *C^i^_n_* is the reference value for heavy metal *i,* which is the background value in Niujiaotang; *E^i^_r_* is the environmental risk index of heavy metal *i*; *T^i^_r_* is the toxicity response coefficient of heavy metal *i* [25]; and *RI* is the multi-element environmental risk composite index. Reference and toxicity coefficient for various heavy metals in Table 1.

#### 2.4.2. Nemeiro Comprehensive Pollution Assessment

The Nemeiro comprehensive pollution index (*P_N_*) highlights the measured degree of soil contamination by the maximum contamination to reflect the overall contamination status of the soil [26]. It is calculated as follows:

(4) $$\begin{array}{c} P_{N}=\sqrt{\frac{P_{imax}^{2}+P_{iavg}^{2}}{2}} \end{array}$$

where *P_imax_* and *P_iavg_* are the maximum and average pollution index values of heavy metal *i* in the soil, respectively.

#### 2.4.3. RAC Method (Risk Assessment Code)

The *RAC* is mainly related to *F*1, and it is calculated as follows:

(5) $$\begin{array}{c} RAC=\frac{C_{F1}}{C_{T}}\times 100\%, \end{array}$$

where *RAC* is the mass fraction of *F*1 (weak acid extracted state) in the total mass, %; *C_F_*_1_ is the concentration of *F*1 (weak acid extracted state), mg·kg^−1^; and *C_T_* is the sum of the four BCR forms, mg·kg^−1^ [22].

#### 2.4.4. RSP Method (Ratio of Secondary Phase to Primary Phase)

The *RSP* method divides the soil into primary and secondary phases, and it evaluates the degree of soil heavy metal contamination by calculating the ratio of the secondary phase (weathering products of primary minerals and exotic secondary materials) to the primary phase [27]. The *RSP* is calculated as follows:

(6) $$\begin{array}{c} RSP=\frac{M_{\;sec\;}}{M_{\;prim\;}}, \end{array}$$

where *M_sec_* is the content of heavy metals in the secondary phase, mg·kg^−1^; and *M_prim_* is the residual state (primary phase) content, mg·kg^−1^.

The above four soil heavy metal risk evaluation methods are widely used in China and other countries, and the industry-accepted evaluation criteria for each method are detailed in Table 2.

### 2.5. Human Health Risk Assessment

The human health risks posed by heavy metals in agricultural products were evaluated using internationally recognized chemical carcinogen and chemical non-carcinogen risk evaluation models. According to the International Agency for Research on Cancer (IARC), the EPA Integrated Risk Information System (IRIS) database, and the classification system compiled by the World Health Organization (WHO), Cd and As are chemical carcinogens, whereas Pb and Zn are non-carcinogens.

The human health risk for a carcinogenic chemical can be calculated as follows:

(7) $$\begin{array}{c} R_{i}=ADD_{i}\times SF_{i} \end{array}$$

where *R_i_* is the individual carcinogenic annual risk attributed to carcinogenic chemical *i*, a^−1^; *ADD_i_* is the average daily exposure dose to the carcinogenic chemical *i*, mg·(kg·d)^−1^; and *SF_i_* is the carcinogenic intensity factor of *i*, (mg/kg/day)^−1^. The grading criteria are as follows: *R_i_* < 1 × 10^−6^ y^−1^ indicates negligible risk to human health; 1 × 10^−6^ < *R_i_* < 1×10^−4^ a^−1^ indicates acceptable risk; and *R_i_* > 1 × 10^−4^ a^−1^ indicates significant risk to human health.

The human health risk for non-carcinogenic chemicals can be calculated as follows:

(8) $$\begin{array}{c} H=\frac{ADD_{i}}{RfD}, \end{array}$$

where *H* is the individual non-carcinogenic annual risk attributed to non-carcinogenic chemicals, y^−1^; and *RfD* is the reference dose of non-carcinogenic chemical exposure, mg·(kg·d)^−1^. The grading criteria are as follows: *H* ≤ 1 indicates that the ingestion of crop products does not pose a health hazard; 1 < *H* ≤ 10 indicates a high health hazard; and *H* > 10 indicates a risk of chronic poisoning [28].

*ADD_i_* was calculated as follows:

(9) $$\begin{array}{c} ADD_{i}=\frac{C_{i}\times IR\times EF\times ED}{BW\times AT\;}, \end{array}$$

where *C_i_* is the content of heavy metal *i* in a crop sample, mg/kg; *IR* is the daily crop consumption per capita (kg/d); *EF* is the annual exposure days to heavy metals, d/y; *ED* is the average exposure period, y; *BW* is the average human body weight, kg; *AT* is the life expectancy, d^1^. The parameters for the health risk evaluation model in Equations (7)–(9) are shown in Table 3.

## 3. Results and Discussion

**Figure 2:**
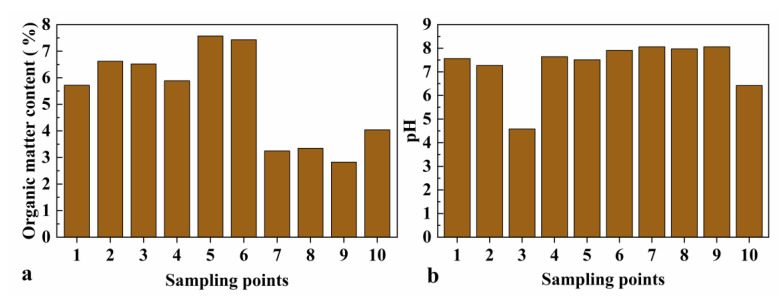
Physical and chemical properties of soil samples. (**a**) Organic matter content of sampling points. (**b**) pH of sampling points. (sampling points: (1, 3, 5, 6) pak choi, (2) rice; (4) spring onion; (7, 10) radish; (8) Chinese cabbage; (9) *Chrysanthemum coronarium*).

**Figure 3:**
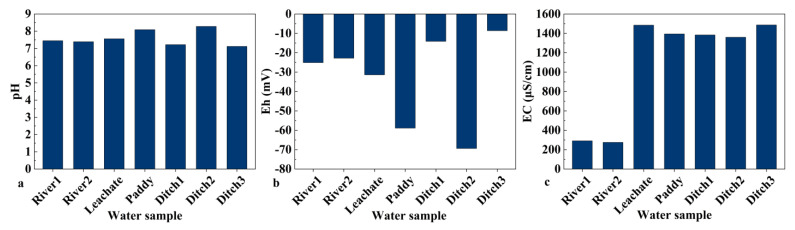
Physical and chemical properties of water samples: (**a**) pH; (**b**) redox potential Eh; (**c**) electrical conductivity EC.

The physicochemical properties of soils affect the biological effectiveness of heavy metals mainly by influencing their morphology [35]. In this context, pH is one of the most important soil physicochemical properties because it affects the activity of heavy metals. Under acidic conditions, heavy metals are more mobile and more easily absorbed by plants [36]. Figure 2b shows that the average soil pH in the study area was 7.298, and that the soil was alkaline in all sample collection points, except point 3. The exposed substratum in the study area is Cambrian Balang Formation shale with a small amount of silt-fine sandstone and a thin layer of tuff in the upper part [37]. Different parent rocks lead to different soil physicochemical properties. Therefore, the difference in parent rocks was considered the main reason for the pH difference between the sampling points. Soil organic matter ranged from 2.822% to 7.572% in Figure 2a. Table 4 shows that the concentrations of heavy metals in the water bodies did not exceed the national surface water quality standard for Class V. From Figure 3, it can be seen that the average value of Eh in the water samples was −32.9 mV (the range of Eh was −8.6 to −69.4); the average value of EC was 1096.571 S·cm^–1^ (the range of EC was 275 to 1487); the average value of pH of the water samples was 7.59 (the range of pH was 7.12 to 8.28), which was basically weakly alkaline. Pan showed that the reason for the alkaline nature of the water in the Oxnard tailing pond mine is mainly related to the neutralizing and buffering effect of carbonate rock on the acidic wastewater produced by sulfide ores [38].

### 3.1. Heavy Metal Contents and Geochemical Characteristics of Agricultural Soils

**Figure 4:**
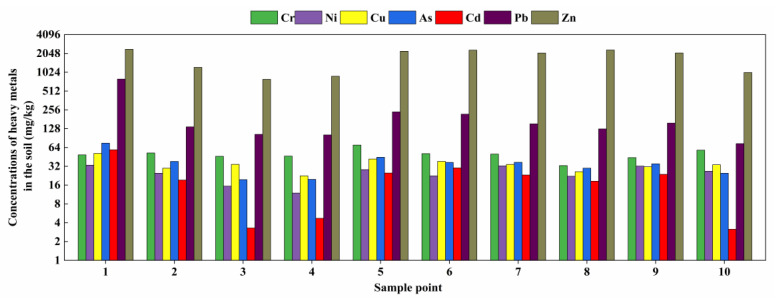
Heavy metal contents in farmlands. Sampling points are the same as in Figure 2.

The contents of seven heavy metals in agricultural soils in the study area are presented in Table 5 and Figure 4. The heavy metal contents in soil samples from different sampling points varied widely. The average concentrations (and ranges) of As, Cd, Pb, and Zn were 36.096 (19.581–75.149) mg/kg, 20.980 (3.145–58.748) mg/kg, 210.503 (73.679~796.233) mg/kg, 1730.283 (788.423~2382.207) mg/kg, which were 2.927, 91.216, 9.144, and 15.346 times the background values of soils in Niujiaotang, and 1.805, 31.787, 5.980, and 17.390 times the background values in Guizhou Province, respectively. Among the heavy metals, Cd exceeded the standard substantially, and Zn, which was the most abundant heavy metal, presented the greatest accumulation.

The content and distribution of heavy metals in soils are related to their sources. Soils near tailings ponds are often contaminated via atmospheric deposition, ground diffusion, and infiltration. Pb–Zn smelting and the associated wastewater sludge are the main sources of As, Cd, Pb, and Zn contamination to nearby areas [39]. The sampling sites in this study were distributed near Pb–Zn mines, and the ancient Zn refining method used in the early years of the Guizhou Zn mines produced pollutants such as Cd, Pb, and Zn, which were released into the atmosphere. In addition, exhaust from motor vehicles on the roads near tailings ponds increases the atmospheric content of heavy metals, such as Cd, Pb, and Zn, which can enter the soil environment through atmospheric deposition [40]. Guizhou is located in the southwest karst region, with a warm and rainy climate and high leaching potential due to weathering. The weathering of rocks releases heavy metals and as such can be a major heavy metal source [41]. Moreover, heavy metals in cultivated soils exceeded the standard values, whereas the concentrations in natural soils were below the background values, which indicated that agricultural cultivation also is a major source of soil heavy metals. Therefore, the soil pollution with As, Cd, Pb, and Zn was attributed to Pb–Zn mining activities, transportation, parent rock weathering, and agricultural farming.

### 3.2. Soil–Plant Heavy Metal Migration and Enrichment Characteristics

#### 3.2.1. Morphological Distribution of Heavy Metals in Agricultural Soils

**Figure 5:**
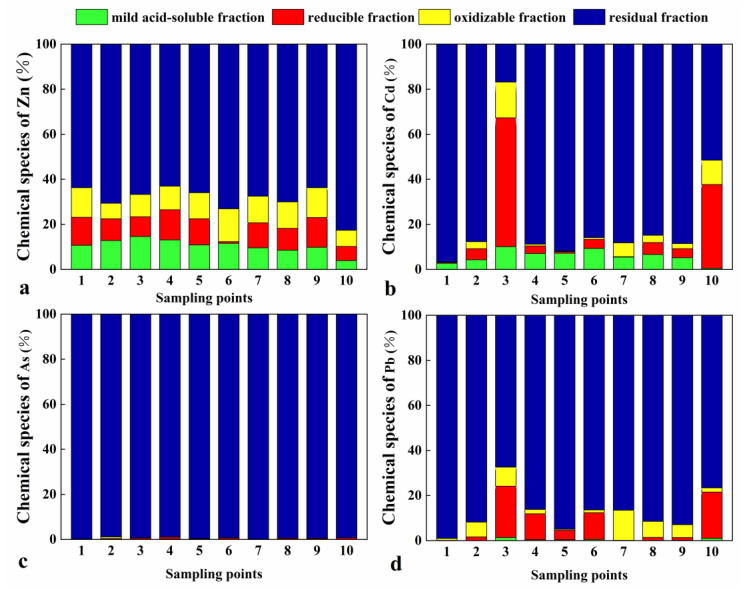
Chemical proportions of four characteristic heavy metals. Chemical species of: (**a**) Zn; (**b**) Cd; (**c**) As; (**d**) Pb. Sampling points are the same as in Figure 2.

Figure 5 shows the average morphological distribution of each element based on morphological analysis of 10 BCR sequentially extracted rhizospheric soil samples. The extraction steps of the chemical forms of heavy metal elements were conducted according to the sequential extraction scheme of the European Community Standards Agency (BCR sequential extraction procedure). The residual state of Zn was the lowest (68.248%), followed by Cd (88.717%). In contrast, the residual states of Pb, As, Cu, Ni, and Cr were all above 90%, which indicated poor migration ability, that is, they are not easily absorbed by organisms and, therefore, are less harmful to the environment. The weak acid extractable states of Zn and Cd accounted for 10.358% and 5.442%, respectively, whereas those of the other elements were less than 1%. The oxidizable state of Zn was 11.737%, whereas those of the other elements were less than 4%. Zn showed various morphological distribution characteristics. The weak acid extractable, reducible, and oxidizable states accounted for similar proportions, with averages of 10.358%, 9.657%, and 11.737%, respectively. The smaller proportion of the reducible state might be related to the redox environment of the soil. [42] have shown that changes in soil redox conditions may cause oxidizable and reducible Zn to be released from the soil. The lowest Cd content was observed for the oxidizable state, which might be related to the low uptake of Cd by organic matter [43].

The biologically effective state of heavy metals is the sum of their first three forms, which are prone to migratory transformation and re-release into the environment and, therefore, pose serious ecological hazards upon plant or animal intake [44]. The order of biologically effective state contents of the heavy metals in this study was Zn (31.75%) > Cd (11.28%) > Pb (7.99%) > Cu (3.02%) > Ni (2.91%) > Cr (1.55%) > As (0.55%). Among them, Zn and Cd presented relatively high proportions of the biologically effective state (>10%), with a high migration capacity and potential hazard. Therefore, in agricultural production, Zn and Cd are more easily absorbed and used by plants.

The content of weak acid-extractable Cd increased with the increase in overall Cd concentration. Nevertheless, pH and other soil physicochemical features affected the Cd content to a higher extent, which is consistent with results reported by [45]).

#### 3.2.2. Heavy Metal Pollution of Crops

Since the farmlands in the study area were contaminated with heavy metals, the crops growing on them also had a potential risk of heavy metal contamination. Table 6 shows that the exceedance rates of heavy metals (Cr, Ni, As, Cd, Pb, Zn) in pak choi near the mine area ranged from 11.015% to 49.985% (refer to GB2762-2017, GB15199-1994, and GB13106-1991). The exceedance rates of the heavy metals in rice ranged within 0.152–48.042%, whereas those in Chinese cabbage ranged from 0.473% to 112.433%. Among the heavy metals, the rate of Cu was exceeded only in *Chrysanthemum coronarium* and pak choi in points 5 and 6, indicating that these crops have a strong Cu enrichment ability. Pb was exceeded in all crops in all points, except for radish in point 10. The concentration of Zn, Cd, and Pb in pak choi were significantly higher than those in the other five crops, and the exceedance rate of Zn in pak choi in point 3 was higher than that in all other crops.

The results showed that the overall exceedance rate in the crops was in the order rice > pak choi > Chinese cabbage > spring onion > *Chrysanthemum coronarium* > radish. Zn had the highest concentration in all six crops, and its average content in descending order was: pak choi > Chinese cabbage > spring onion > rice > radish > *Chrysanthemum coronarium*, which may be related to the growth habits of the crops. Zn is an essential plant element, which can explain the highest effective content of Zn and its easy absorption by plants [46]. The difference between the mean contents of Cr and As was not large, and As > Cr for all crops except pak choi in sites 5 and 6 and rice in site 2. The effective state of As accounted for the lowest proportion of total As, but the content of As in the crops was higher than that of Cr, indicating that the crops in this study presented a higher absorption rate for As than for Cr. Moreover, Cd exceeded the standard in all crops because it is easily absorbed by crop plants [47].

There was no clear pattern in the concentration of Cu, Cr, and As in the different crops. The generally low content of Ni was attributed to its low content in the soils. The mean concentration of Pb was high in soils, but generally low in crops because Pb has relatively weak mobility and does not easily accumulate in plants [48,49]. The Cr concentration was generally low in soils, but often exceeded the standard in crops, especially in pak choi, and this was attributed to its strong soil mobility [50,51]. Contamination with heavy metals such as Cd, Zn, and As can substantially inhibit the growth and development of crop plants and animals, and long-term ingestion by local residents through dietary exposure can pose a considerable health risk [52]. Therefore, the cultivation of pak choi and rice, which presented high heavy metal enrichment capacity, should be avoided in the area, and radish and spring onion should be given preference so as to reduce the human health risks.

#### 3.2.3. Enrichment and Transport of Heavy Metals

The enrichment factor is an indicator of heavy metal enrichment in crops, and the ratio of the heavy metal content in the edible parts of a crop to that in the soil is used to analyze the soil heavy metal uptake and cumulative effect of the crop [53]. Larger enrichment factor values indicate that crops are more likely to uptake the heavy metal from the farmland, that is, they present greater biological effectiveness [54]. The metal enrichment coefficients of the edible part of rice, a typical crop near the tailings area, followed the order Cr (1.643) > As (0.172) > Cu (0.138) > Ni (0.137) > Zn (0.020) > Cd (0.010) > Pb (0.005), which indicated that the enrichment capacity of the edible part of rice for Cr in the environment was significantly higher than that for other heavy metals. The enrichment factor values of Zn, Cd, and Pb were all less than 0.1, indicating weak enrichment ability.

The transport coefficient is the ratio of the concentration of a heavy metal in the aboveground part of a crop to that in the roots. It can reflect the migration and transfer capacity of heavy metals from the roots to the aboveground part of plants [55]. The transport coefficients for heavy metals from root to edible part of rice were in the order As (0.316) > Cr (0.280) > Cu (0.159) > Ni (0.088) > Zn (0.071) > Pb (0.014) > Cd (0.013), which indicated that the transport capacity for As from root to edible part in rice was higher than that for other heavy metals.

### 3.3. Ecological Environment and Human Health Risk Assessment

#### 3.3.1. Ecological Risk Assessment

**Figure 6:**
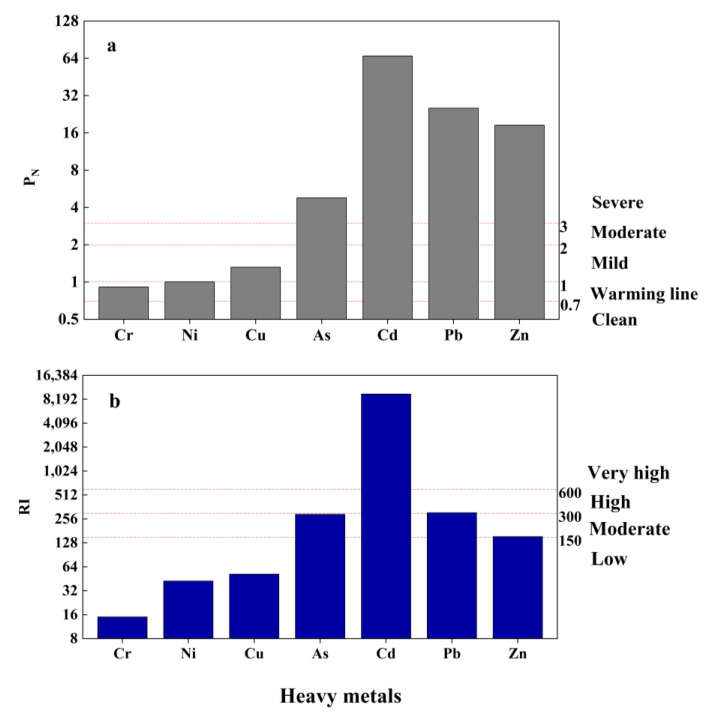
Nemeiro comprehensive pollution assessment and potential ecological risk assessment of soils. (**a**) P_N_ of heavy metals; and (**b**) RI of heavy metals.

The results of evaluation of the soil environment near the Pb–Zn mining area using the potential ecological risk and Nemeiro indices are shown in Figure 6. The single factor contamination index values of soil heavy metals followed the order Cd > Zn > Pb > As > Cu. Thus, the agricultural soil near the Pb–Zn mining area was severely contaminated with Cd and Zn, which presented mean single factor contamination index values of 91.216 and 15.346, respectively. Pb, As, and Cu presented single pollution index values > 1, with different degrees of pollution.

According to Nemeiro comprehensive pollution assessment, As, Cd, Pb, and Zn pollution was high in 10 sampling sites, and the Nemeiro index values followed the order Cd > Pb > Zn > As > Cu > Ni.> Cr. While Ni and Cu presented slight exceedances, other heavy metals greatly exceeded the standard values. The highest Nemeiro index was recorded in sampling point 1, which is located near the leachate flow path of the Pb–Zn mine, and the highest concentrations of Cd, Pb, and Zn were also noted in point 1, which may be the main reason for the high Nemeiro index at this site.

The potential ecological index of heavy metals followed the order Cd > Pb > As > Zn > Cu > Ni > Cr, with As and Zn posing medium risk, Pb posing high risk, and Cd posing very high risk. Therefore, the potential environmental impacts of As, Cd, Pb, and Zn should be carefully considered as they might be considerably high. Therefore, mitigation and management strategies should be developed and adopted to mitigate and prevent potential risks. Points 3, 4, and 10 were at moderate ecological risk, and the remaining seven points were at very high ecological risk, among which points 1 and 10 were at the highest and lowest risk, respectively.

**Figure 7:**
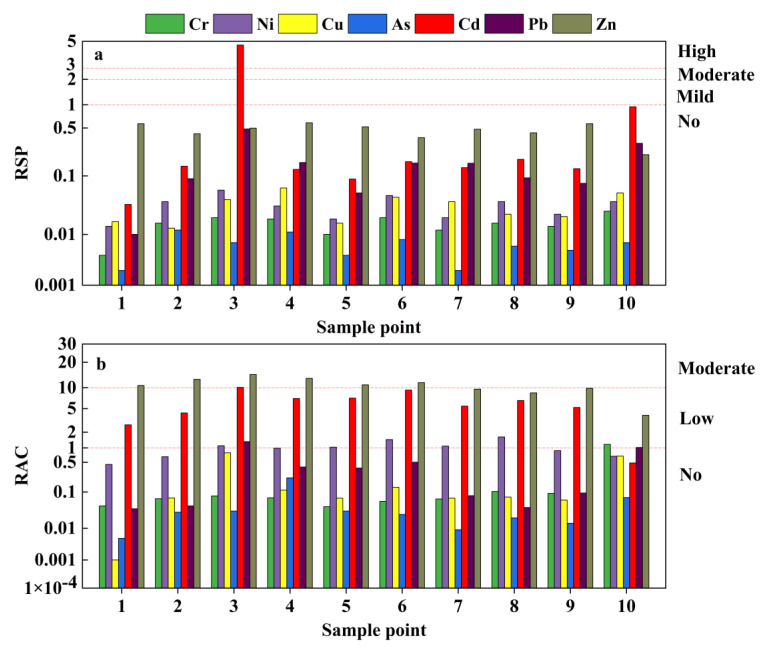
(**a**) Ratio of secondary phase to primary phase (RSP); and (**b**) risk assessment coding (RAC) evaluation results of soil samples.

The results of soil heavy metal pollution risk evaluation based on RAC and RSP are shown in Figure 7. The results of the RSP method (refer to Table 2) indicated that only Cd in soils near the tailings pond (point 3) was associated with heavy pollution, and the proportion of its biologically effective state was 83.19%, which represented a substantial threat to the surrounding environment. The other heavy metals were not associated with pollution levels, and Cr, Ni, Cu, and As were observed mainly in the stable residue state, which represents a lower potential threat to the environment. According to the RAC results, Zn posed a medium risk in 60% of the sampling points, namely in those near tailings, and low risk in the remaining points. Cd posed medium risk at point 3, and low risk in 80% of the points. Pb exerted no risk in 80% of the points. Ni posed low risk in 50% of the sampling points. Cr was only at low risk at point 10, the rest of the points were at no risk, whereas Cu and As presented no risk. The risk levels of the seven heavy metals in the farmlands were in the order Zn > Cd > Ni > Pb > Cu > Cr > As, which is consistent with the conclusion that Pb, As, Cu, Ni, and Cr have the highest residual state and poor migration ability. Together, Zn and Cd posed the greatest environmental threats.

The binding strength of different forms of heavy metals to the fixed medium varies, and different forms also present variability in chemical behavior and toxicity. Lower binding strength and weaker mobility lead to higher environmental risks [56]. The distribution characteristics of different forms of heavy metals in the environment can help estimate their toxicity, mobility, and potential ecological risks [57]. When total and morphological contents of heavy metals are used to assess environmental pollution and ecological risks, different specific values in the total evaluation method can lead to high variability of the results.

It is reported that an increase in the total heavy metal concentration was associated with an increase in the stable metal content (i.e., the residual state) [58]. However, the effect on active states, such as the exchangeable state, was minimal. Therefore, the exchangeable and carbonate-binding states should be carefully examined when evaluating ecological risk, as they have strong migration and toxicity. Evaluation of the states of heavy metals provides a more objective and accurate analysis of the potential ecological risk of pollutants to the environment.

#### 3.3.2. Health Risk Assessment

**Figure 8:**
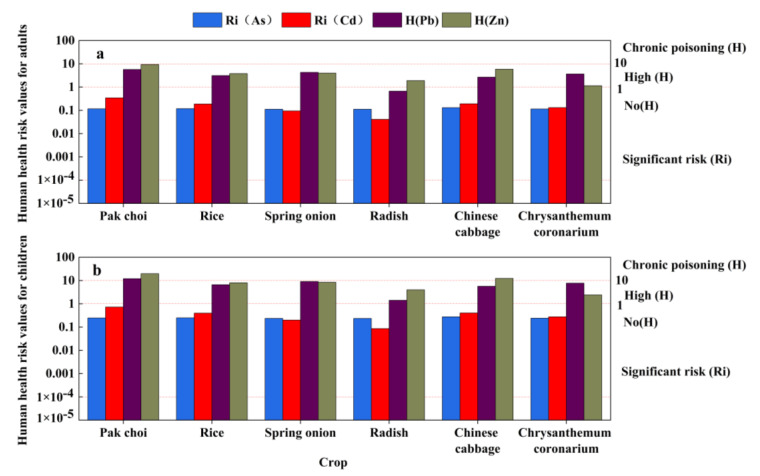
Human health risk values of As, Cd, Pb and Zn in six crops for: (**a**) adults; and (**b**) children.

Heavy metals in the soil can be absorbed by crop plants through the roots and accumulate in different organs and tissues. If the edible parts accumulate large amounts of heavy metals, they can cause serious human health effects via the food chain [59]. The risks of carcinogenic chemicals (Cd, As) and non-carcinogenic chemicals (Pb, Zn) in six crops to the health of adults and children are shown in Figure 8. The results show that the risks of As and Cd in the six crops were higher than the maximum acceptable risk level of 1 × 10^−4^ for both adults and children, which indicated that the accumulated As and Cd posed serious health risks. The intake of Pb through pak choi by children exceeded the maximum acceptable risk level and posed a risk of chronic toxicity. The risks of Pb to both adults and children were lower than 10 (except for the intake of radish by adults, which presented a low risk), but the possibility of negative effects was high. The health risks of Zn in pak choi and Chinese cabbage for children were above the maximum acceptable risk levels, and Zn in the other crops (below 10 for adults and children) presented a high possibility of health risks for children and adults.

## 4. Conclusions

The average pH of the investigated agricultural soils was 7.298 (4.58–8.06), which indicated weak alkalinity. The organic matter ranged within 2.822–7.572%, which indicated a low soil fertility. The concentrations of heavy metals in the Caiyuan River and leachate met the standard of surface water class V. The average pH of the water body was 7.587, and Eh and EC ranged from −69.4 to −8.6 mV and from 275 to 1487 μS·cm^–1^, respectively.

In farmlands, Cd exceeded the standard more substantially than the other metals, and Zn presented the highest abundance and accumulation and thus, the most evident pollution (maximum concentration of 2382.207 mg/kg). The average concentrations of As, Cd, Pb, and Zn were 2.927, 91.216, 9.144, and 15.346 times the soil environmental background values in Niujiaotang, and 1.805, 31.787, 5.980, and 17.390 times the soil environmental background values in Guizhou Province, respectively. The extraction steps of the chemical forms of heavy metal elements were conducted according to the sequential extraction scheme of the European Community Standards Agency (BCR sequential extraction procedure). The proportions of Zn states in agricultural fields were in the order residue > oxidizable > reducible > weak acid extractable state, whereas those of Cd states were in the order residue > weak acid extractable > reducible > oxidizable state.

The degree of heavy metal contamination of crops followed the order pak choi > Chinese cabbage > spring onion > rice > radish > *Chrysanthemum coronarium*, and the degree of individual heavy metal contamination of crops was in the order Zn > As > Cr > Cd > Ni > Pb. The highest content of Zn in the crops might be related to its higher soil concentration relative to other metals. Except for Cu, all heavy metals exceeded the limits of China’s food hygiene standards. Carcinogenic chemicals (Cd, As) and non-carcinogenic chemicals (Pb, Zn) in six crops posed significant health risks to adults and children, and Zn and Pb in pak choi and Zn in Chinese cabbage posed a risk of chronic poisoning to children. The enrichment capacity of the edible part of rice for Cr was significantly higher than that for the other heavy metals, and the transport capacity from the root system to the edible part of rice was higher for As than that for the other heavy metals.

Comparative analysis of the results of the four selected methods revealed that the farmlands were severely polluted. The Nemeiro index values were in the order Cd > Pb > Zn > As > Cu > Ni > Cr, and the potential ecological index values followed Cd > Pb > As > Zn > Cu > Ni > Cr. The RAC and RSP evaluation results showed that, together, farmland and crops had a high degree of pollution and posed a high ecological risk. The discrepant results obtained from evaluations based on total or morphological heavy metal contents indicated that the method based on morphological contents can more objectively and accurately evaluate the potential ecological risks of pollutants to the environment. Samples should be collected at different times to study the spatial and temporal distribution, and further studies on the migration transformation of heavy metals between the tailings pond-soil-crop should be conducted.

## Figures and Tables

**Table 1:** Reference (*C^i^_n_*) and toxicity coefficient (*T^i^_r_*) for various heavy metals.

Element	*C^i^_n_* (mg/kg)	*T^i^_r_*
As	12.33	10
Cd	0.23	30
Cr	66.6	2
Cu	33.18	5
Ni	29.39	5
Pb	23.02	5
Zn	112.75	1

**Table 2:** Evaluation criteria of the four methods for the evaluation of heavy metals in the soil.

Method	Risk Levels					Literature
Potential ecological risk index (RI) method	RI < 150	150 ≤ RI < 300	300 ≤ RI < 600	600 ≤ RI	/	[23]
	Low	Moderate	High	Very high	/	
Nemeiro index method	P_N_ ≤ 0.7	0.7 < P_N_ ≤ 1	1 < P_N_ ≤ 2	2 < P_N_ ≤ 3	P_N_ > 3	[26]
	Clean	Warning Line	Mild	Moderate	Severe	
Risk assessment code (RAC)	RAC < 1%	RAC < 10%	10% ≤ RAC < 30%	30% ≤ RAC < 50%	≥50%	[22]
	No	Low	Moderate	High	Very high	
Ratio of secondary phase to primary phase (RSP)	RSP ≤ 1	1 < RSP ≤ 2	2 < RSP ≤ 3	RSP > 3	/	[27]
	No	Mild	Moderate	High	/	

**Table 3:** Parameters in the human health risk assessment model.

Parameter	Adults	Children	Source
IR	0.355	0.233	[29]
EF	350	350	[30]
ED	24	6	
BW	61.8	19.2	
AT	ED × 365	ED × 365	[31]
RfD	Pb = 0.0035		[32]
	Zn = 0.3		[33]
SF	As = 1.5; Cd = 6.1		[34]

**Table 4:** Concentrations of heavy metals in water samples and respective national standard values for Class V surface water (mg/kg).

Heavy Metals	Cr	Ni	Cu	Zn	As	Cd	Pb
River 1	4.579	0.233	0.331	17.45	0.445	N.D	0.272
River 2	3.659	0.026	1.198	6.834	0.372	N.D	0.298
Leachate	3.146	4.73	2.215	49.82	0.051	N.D	0.241
Paddy water	4.051	3.292	1.996	100.1	0.053	0.278	0.278
Ditch 1	3.914	3.64	1.888	253.3	0.048	0.208	0.290
Ditch 2	3.6	2.833	1.839	134.4	N.D	0.31	0.248
Ditch 3	3.8	3.339	1.834	42.08	0.95	N.D	0.328
Surface water standard (Class V)	100	20	1000	200	100	10	100

**Table 5:** Contents of heavy metals in the studied soils (mg/kg).

Type of Value	Cr	Ni	Cu	As	Cd	Pb	Zn
Maximum	69.99	33.40	51.34	75.15	58.75	796.22	2382.21
Minimum	32.83	11.93	22.52	19.58	3.15	73.68	788.42
Average	50.00	24.97	34.40	36.10	20.98	210.50	1730.28
Median	49.47	25.71	34.06	35.95	21.24	144.91	2078.39
Standard deviation values	9.10	6.85	7.72	15.20	15.63	201.09	630.02
Content in tailings	1183.37	4328.82	346.03	30.64	69.25	200.93	5470.01
Background in Niujiaotang	66.60	29.39	33.18	12.33	0.23	23.02	112.75
Background in Guizhou Province	95.90	39.10	32.00	20.00	0.66	35.20	99.50

**Table 6:** Heavy metal concentrations in crops (mg/kg).

Crop	Sampling Point	Cr	Ni	Cu	As	Cd	Pb	Zn
Pak choi	1	2.974	2.614	4.068	13.609	9.277	0.663	323.529
	3	2.052	1.911	4.864	13.002	5.184	1.714	779.324
	5	17.962	7.639	12.316	17.778	15.065	11.441	618.290
	6	62.525	16.899	13.106	11.958	11.262	0.599	305.666
Rice	2	49.042	21.713	3.443	14.230	5.561	1.982	206.058
Spring onion	4	2.625	0.870	5.160	13.435	2.797	2.723	216.351
Chinese cabbage	8	2.997	2.663	4.691	15.736	5.672	1.700	317.910
*Chrysanthemum coronarium*	9	2.846	1.531	12.994	13.793	3.891	2.311	62.066
Radish	7	2.142	1.517	3.076	14.796	1.631	0.594	181.095
	10	2.137	1.678	0.706	12.016	0.791	0.255	24.330

## Data Availability

The authors declare that data supporting the findings of this study are available within the article.
